# UPLC-QTOF MS-Based Serum Metabolomic Profiling Analysis Reveals the Molecular Perturbations Underlying Uremic Pruritus

**DOI:** 10.1155/2018/4351674

**Published:** 2018-01-10

**Authors:** Qiong Wu, Huan Zhang, Jia-rong Ding, Zhan-ying Hong, Hao Wu, Zhen-yu Zhu, Zhi-yong Guo, Yi-feng Chai

**Affiliations:** ^1^School of Pharmacy, Second Military Medical University, Shanghai 200433, China; ^2^Department of Nephrology, Changhai Hospital, Second Military Medical University, Shanghai 200433, China; ^3^Analysis and Measurement Center, School of Pharmacy, Second Military Medical University, Shanghai 200433, China

## Abstract

As one of the most troublesome complications in patients with chronic renal disease, the etiology of uremic pruritus remains unknown, and the current therapeutic approaches are limited and unsatisfactory. To identify potential biomarkers for improving diagnosis and treatment and obtain a better understanding of the pathogenesis of uremic pruritus, we compared serum metabolome profiles of severe uremic pruritus (HUP) patients with mild uremic pruritus (LUP) patients using ultraperformance liquid chromatography-quadruple time-of-flight mass spectrometry (UPLC-QTOF MS). Partial least squares discriminant analysis (PLS-DA) showed that the metabolic profiles of HUP patients are distinguishable from those of LUP patients. Combining multivariate with univariate analysis, 22 significantly different metabolites between HUP and LUP patients were identified. Nine of the 22 metabolites in combination were characterized by a maximum area-under-receiver operating characteristic curve (AUC = 0.899) with a sensitivity of 85.1% and a specificity of 83.0% distinguishing HUP and LUP. Our results indicate that serum metabolome profiling might serve as a promising approach for the diagnosis of uremic pruritus and that the identified biomarkers may improve the understanding of pathophysiology of this disorder. Because the 9 metabolites were phospholipids, uremic toxins, and steroids, further studies may reveal their possible role in the pathogenesis of uremic pruritus.

## 1. Introduction

Uremic pruritus (UP) is one of the most common and uncomfortable symptoms in chronic kidney disease (CKD) patients, especially in hemodialysis patients [1]. The prevalence of uremic pruritus is quite high and is reported in approximately 40% to 50% of CKD patients, and it has a significant association with a lower quality of life, poor sleep, depression, and increased mortality [2]. Although many studies have demonstrated that many factors are related to the occurrence of UP, the etiology and pathophysiology of uremic pruritus have not yet been found [3–5]. Over the past few decades, there have been a variety of traditional causes of treatment, but there is no specific treatment for patients with UP and many of the available therapeutic modalities are not satisfactory [6].

As an alternative approach for biomarker discovery, metabolomics (or metabolite profiling) enables the identification of small-molecule metabolites in biofluids and tissues that are sensitive to altered pathology [7]. Over the past several years, ultraperformance liquid chromatography coupled to time-of-flight mass spectrometry (UPLC-MS), which is an information-rich analytical technique, has become an advanced and useful tool [8]. Compared to other biomarker approaches, metabolomics might provide more insight into pathogenesis [9]. Importantly, serum tests based on metabolic profiles are relatively inexpensive, rapid, and automated. Although metabolomics has been widely used in molecule discovery for early diagnosis for UP, disease detection, targeted therapy, and drug response [7], no studies have been performed leading to biomarker discovery for early diagnosis for UP in CKD patients.

We hypothesize that there are specific biomarkers that may be detected in the serum of uremic pruritus patients. To identify potential biomarkers for the noninvasive diagnosis of uremic pruritus, we conducted a UPLC-QTOF MS-based serum metabolomics analysis for uremic pruritus patients and used multi- and univariate statistical analyses of the metabolome data to identify specific biomarkers for uremic pruritus. The diagnostic performances of the identified biomarkers were evaluated using receiver operating characteristic (ROC) curve analysis. The study used the method previously published by our group [10, 11]. In this pilot study, metabolic profiling of serum sample was conducted to explore potential diagnostic biomarkers for uremic pruritus and improve the understanding of pathogenesis in this disorder and the patients' quality of life.

## 2. Materials and Methods

In this study, we followed the methods previously published by our group [10, 11].

### 2.1. Study Group

Two hundred uremic patients who needed hemodialysis were recruited. A visual analogue scale (VAS) measuring the general severity of pruritus from 0 (no itch) to 10 (maximum imaginable itch) was used to measure the severity of itching during the last 3 days [12, 13]. The mild pruritus group (LUP) included 47 patients (VAS score of 0–3), the moderate pruritus included 81 patients (VAS score of 3–7), and the severe and very severe pruritus group (HUP) contained 72 patients (VAS score of 7–10). Selected mild pruritus and severe pruritus groups participated in the experiment. Venous blood samples were obtained from patients recruited at the Department of Nephrology, Changhai Hospital of the Second Military Medical University. The patient characteristics are shown in Table 1. Written consent was collected from all of the patients who participated in this study. The protocol of the study and the procedures designed for sample collection were reviewed and approved by the ethical committee of the Second Military Medical University, Shanghai, China.

### 2.2. Sample Collection and Preparation

Blood sample collection from the patient was done on the same day. Parameters, including gender, age, duration of HD, and haemoglobin, serum albumin, transferrin saturation (TSAT), serum ferritin (fer), creatinine, corrected calcium, phosphate, total cholesterol, triglyceride, and iPTH levels, from each patient were recorded at the time of sampling. Venous blood was collected into a 5 mL vacutainer tube containing the chelating agent ethylene diamine tetraacetic acid (EDTA). The tube was centrifuged at 3000 rpm for 15 minutes. The supernatant (serum sample) was aliquoted and stored at −80°C until analysis. No sample underwent more than two freeze-thaw cycles prior to LC-MS analysis.

The serum samples (100 *μ*L) were thawed at 4°C followed by the addition of 400 *μ*L methanol/acetonitrile (1 : 1/v : v). The mixture was then vortexed vigorously for 30 s followed by centrifugation at 14000 ×g for 15 min at 4°C. The supernatant (50 *μ*L) was transferred to an autosampler vial and an aliquot of 4 *μ*L was injected for LC-MS analysis.

### 2.3. Global Metabolite Profiling

In this study, using the method previously published by our group [10], the UPLC-QTOF/MS analyses were performed on an Agilent 1290 Infinity LC system configured with an Agilent 6530 accurate-mass quadrupole time-of-flight (QTOF) mass spectrometer (Agilent, Palo Alto, USA). An ACQUITY UPLC HSS T3 column (2.1 mm × 100 mm, 1.8 *μ*m, Waters, Milford, MA, USA) was used. The serum samples were separated at 45°C with a flow rate of 0.4 ml/min. The mobile phase was water with 0.1% formic acid (A) and methanol with 0.1% formic acid (B). The gradient program was as follows: 100% A (0–2 min), 100%–85% A (2–10 min), 85%–70% A (10–14 min), 70%–5% A (14–17 min), 5% A (17–19 min), and 5%–100% A (19-20 min), followed by a 5-minute column reequilibration.

The MS experiments were performed on an Agilent 6530 accurate-mass quadrupole time-of-flight (QTOF) mass spectrometer (Agilent, Santa Clara, CA, USA). The cone gas was nitrogen with a flow of 11 L/h. The following detection parameters were used: fragment voltage, 120 V; capillary voltage, 3.5 kV; gas temperature, 350°C; and source temperature, 120°C. To guarantee mass accuracy and reproducibility, the full MS scan mode was monitored at the mass range of 50–1000* m/z*. In the analyzing process, 10 mM purine (*m/z* 121.0508) and 2 mM hexakis phosphazene (*m/z* 922.0097) were used as internal standards. The centroid data were collected from the instrument. Subsequently, a MS/MS experiment was performed and the experiment parameters were set as follows: MS spectrum acquisition rate, 2 spectra/s; MS/MS spectrum acquisition rate, 0.5 spectra/s; and medium isolation window, 4* m/z*; and collision energy, 20 V.

### 2.4. Data Handing

For data processing, we used the method previously published by our group [14].

## 3. Results

### 3.1. Study Groups and Their Characteristics

Between September 2014 and December 2015, 200 eligible hemodialysis (HD) patients who met the inclusion and exclusion criteria were enrolled in this prospective study; of these patients, 72 were diagnosed as HUP and 47 were LUP, based on their VAS scores. The demographic and clinical characteristics of the prospective cohort are shown in Table 1. The baseline characteristics were comparable in each group. The results showed that the parameters were not significantly different between HUP and LUP, except for the serum albumin and iPTH levels.

### 3.2. Serum Metabolic Profiles

**Figure 1:**
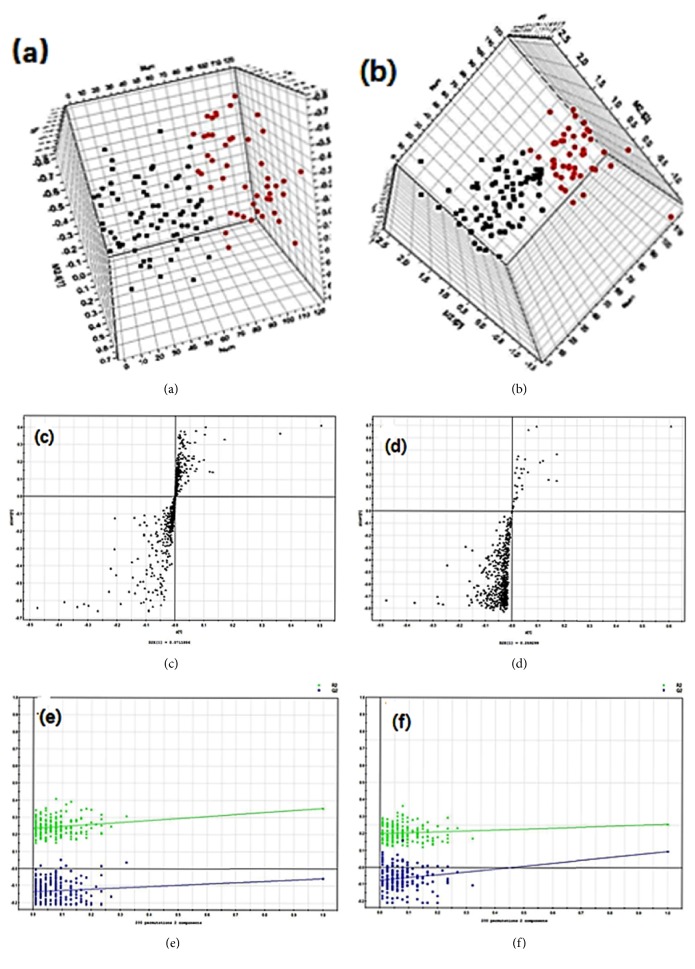
Multivariate data analysis. (a) PLS-DA score map for the HUP and LUP patients in positive mode; (b) PLS-DA score map for the HUP and LUP patients in negative mode; (c) S-plot of the PLS-DA model in positive mode; (d) S-plot of the PLS-DA model in negative mode; (e) validation plot obtained from 200 permutation tests in positive mode; (f) validation plot obtained from 200 permutation tests in negative mode.

There is a clear separation trend between HUP and LUP (Figures 1(a) and 1(b)). In order to validate the model, we performed 200 iterations of permutation testing. These permutation tests compare the advantages of the original model fitting and the fitting of the randomly permuted model. As shown in Figures 1(e) and 1(f), the verification diagram shows that the original model is valid. The criterion of validity is as follows: all* R*2 (cum) and* Q*2 (cum) values on the left are lower than the values on the right, while the blue regression line of* Q*2 (cum) points has a negative intercept.

### 3.3. The Discovery and Identification of Metabolic Biomarkers

**Figure 2:**
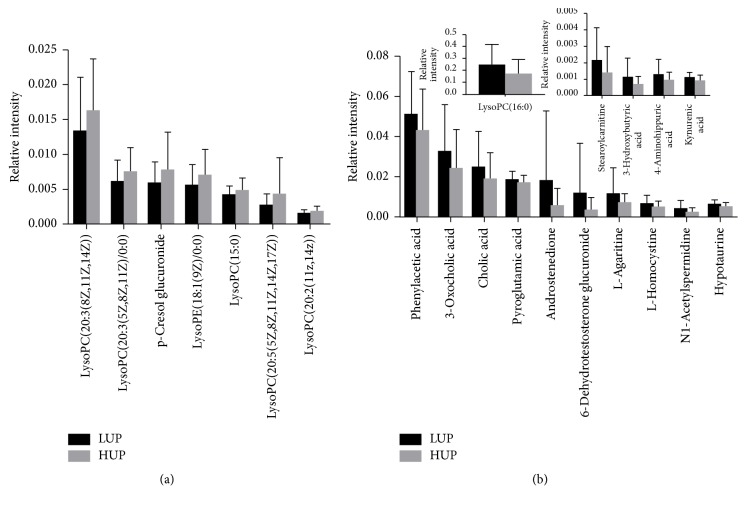
Bar plots showing fluctuations in relative signal intensities of potential biomarkers for HUP and LUP patients.

Metabolites were carefully screened before being approved as potential biomarkers. First, significant original variables were extracted from the S-plot, which is a covariance-correlation-based procedure, thereby reducing the risk of false positives in metabolite selection. The S-plot (Figures 1(c) and 1(d)) derived from the first component of the combined model explained most of the variables in the dataset, in which the ions farthest away from the origin contributed significantly to the clustering of the two groups and may thus be considered potential biomarkers. Next, the variable importance for projection (VIP), reflecting the importance of variables, was applied to filter the important metabolites in the model (VIP ≥ 1). Unpaired Student's *t*-tests were performed as the final testing procedure, and the critical *p* value was set to 0.05 for significantly differential variables. Following the criterion above, 22 metabolite ions (as shown in Table 2) were selected as potential biomarkers related to uremic pruritus. In addition, the bar plots for the relative intensity of 22 potential biomarkers are given in Figure 2.

### 3.4. Diagnostic Performance of Metabolites Identified in Uremic Pruritus

To further validate the potential diagnostic effectiveness of these metabolite signatures, the receiver operating characteristic curve (ROC curve) was plotted individually using the relative intensities of these metabolites (data not shown). Stepwise regression analysis was used to screen the optimal metabolites in combination. LysoPE(20:3(5Z,8Z,11Z)/0:0), LysoPC(20:2(11Z,14Z)), LysoPC(16:0), p-cresol glucuronide, phenylacetic acid, hypotaurine, 4-aminohippuric acid, kynurenic acid, and androstenedione, belonging to phospholipids, uremic toxins, and steroids, were identified as potential biomarkers for uremic pruritus.

**Figure 3:**
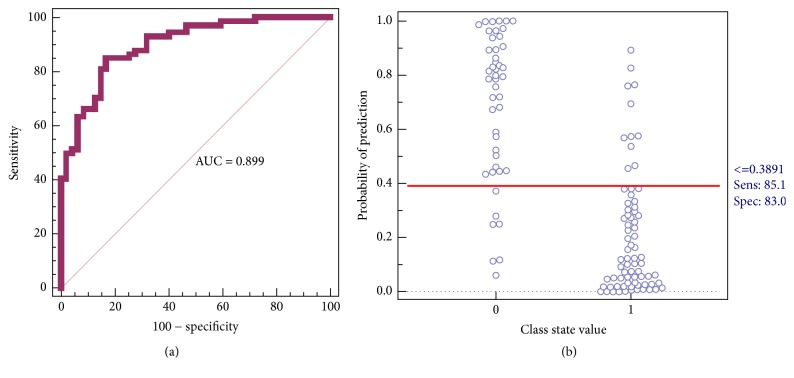
(a) ROC curves based on the binary logistic regression model using the combination of nine serum metabolites; (b) their prediction plots based on the optimal cutoff value obtained from the ROC curves.

Figure 3(a) shows the prediction results using the model constructed by the nine candidate markers for the two groups. Binary logistic regression was used to combine the nine variables into a multivariable. The results indicated that a panel of nine metabolites generated an AUC of 0.899, with a sensitivity of 85.1% and a specificity of 83.0% for distinguishing HUP and LUP (Figure 3(b)). According to the highest prediction sensitivity (85.1%) and specificity (83.0%) of the ROC curves, an optimal cutoff value of 0.3891 was obtained. Based on this cutoff value, it was found that 61 of the 72 samples (84.7%) were correctly classified as HUP. This finding indicated that this simplified serum metabolite signature was a “good” classifier of HUP and LUP patients.

## 4. Discussion

In this study, we present a metabolomics approach for screening potential biomarkers related to UP. By applying UPLC-QTOF MS technology and multivariable statistical analysis methods, 22 significantly different metabolites between HUP patients and LUP patients were identified, and, through stepwise regression analysis, 9 of the 22 metabolites (LysoPE(20:3(5Z,8Z,11Z)/0:0), LysoPC(20:2(11Z,14Z)), LysoPC(16:0), p-cresol glucuronide, phenylacetic acid, hypotaurine, 4-aminohippuric acid, kynurenic acid, and androstenedione) in combination were characterized by a maximum area-under-receiver operating characteristic curve (AUC = 0.899), with a sensitivity of 85.1% and a specificity of 83.0% for distinguishing HUP and LUP. Therefore, these nine compounds, which are phospholipids, uremic toxins, and steroids, can be further investigated to reveal their possible roles in the pathogenesis of UP and to help diagnose UP.

In the present study, the pattern of uremic toxins was disturbed in UP patients, which is consistent with the literature. Uremic syndrome is characterized by the retention of various solutes that would normally be excreted by the kidneys [15]. These uremic solutes have been reported to activate itch fibers, including the profound changes that occur with hyperparathyroidism-associated metabolic bone disease, increased systemic inflammation, and structural alterations in the skin related to dehydration and immune dysregulation of uremia [16]. These factors have been suggested to be possible underlying causes of UP [17, 18]. Therefore, these significantly changed uremic toxins may serve as a triggering factor for UP and need to be further investigated to reveal their detailed mechanism of action.

LysoPE(20:3(5Z,8Z,11Z)/0:0), LysoPC(20:2(11Z,14Z)), and LysoPC(16:0) are phospholipids, which suggested that perturbations of phospholipid metabolism are involved in the pathogenesis of UP. Previous studies have shown that LysoPCs can induce nephrotoxicity through oxidative stress [19]. Furthermore, abundant evidence indicates that LysoPCs induce multiple proinflammatory activities, including stimulating monocytes/macrophages to produce IL-1*β*, the generation of reactive oxygen species, and the promotion of cell growth migration [20, 21]. There are also studies showing that LysoPC is a chemoattractant to T lymphocytes and monocytes, which all play an important role in inflammation in the skin [22, 23]. In addition, the serum P concentration, as a factor in UP, was reported to be associated with increased LysoPC concentrations [24]. Therefore, abnormal phospholipid metabolism may be involved in the pathogenesis of UP by inducing inflammation and increasing the serum P concentration.

Steroids such as androstenedione are also downregulated in UP patients; however, the observation of lower levels of androstenedione in HUP compared to LUP is not completely understood. Although further functional work is needed, we posit one hypothesis that may explain this observation. Androstenedione is the precursor of testosterone, which is an anabolic steroid and is involved in the growth of muscle, bone, and body hair [25]. Both androstenedione and testosterone can be aromatized to estrogen, which are also responsible for bone age maturation [26]. Therefore, decreased androstenedione may indicate abnormal bone function in uremic patients, which is reported to be the cause of UP.

## 5. Conclusions 

The present study indicates that serum metabolome profiling might serve as a promising approach for the treatment of UP and that the identified biomarkers may improve the understanding of pathophysiology of this disorder. Further studies are warranted regarding their possible role in the pathogenesis in UP.

## Figures and Tables

**Table 1:** Demographic description of HD patients with UP.

Parameters	LUP	HUP	*p* value
Age, years	57.34 ± 13.41	60.05 ± 16.91	NS
Male/female	24/23	42/30	--
Dialysis, months	3.70 ± 0.52	3.69 ± 0.44	NS
Hb, g/l	117.06 ± 14.84	101.21 ± 15.39	<0.001
*Kt*/*V*	1.38 ± 0.32	1.30 ± 0.36	NS
TSAT, %	24.16 ± 11.92	22.23 ± 10.41	NS
Fer, *µ*g/l	128.09 (73.63–263.8)	152.58 (74.68–280.51)	NS
iPTH, pg/ml	226 (181.5–399.2)	300.7 (145.4–539)	<0.001
Calcium, mmol/L	2.46 ± 0.26	2.45 ± 0.27	NS
Serum albumin, g/L	39.06 ± 3.33	40.49 ± 2.78	NS
Creatinine, mmol/L	968.70 ± 260.27	1070.93 ± 645.25	NS
Phosphate, mmol/L	1.78 ± 0.50	1.94 ± 0.67	NS
Total cholesterol, mmol/L	3.83 ± 0.87	4.05 ± 1.08	NS
Triglyceride, mmol/L	2.05 ± 1.32	2.12 ± 2.14	NS

Data are expressed as the mean ± SD or as median (first and third quartile), as appropriate.

**Table 2:** Summary of the potential biomarkers related to UP.

Number	*m*/*z*	TR (min)	Adduct	Metabolites	Formula	VIP	*p* value
(1)	424.34	10.14	M + NH4	3-Oxocholic acid	C24H38O5	5.27	0.03
(2)	137.05	1.02	M + H	Phenylacetic acid	C8H8O2	5.19	0.04
(3)	426.36	10.66	M + NH4	Cholic acid	C24H40O5	4.51	0.04
(4)	290.16	3.12	M + Na	L-Agaritine	C12H17N3O4	4.14	0.03
(5)	568.34	10.31	M + Na	LysoPC(20:3(8Z,11Z,14Z))	C28H52NO7P	2.94	0.04
(6)	269.09	1.42	M + H	L-Homocysteine	C8H16N2O4S2	2.82	0.03
(6)	267.07	1.42	M − H	L-Homocysteine	C8H16N2O4S2	1.75	0.02
(7)	205.16	0.64	M + NH4	N1-Acetylspermidine	C9H21N3O	2.67	0.01
(8)	110.01	0.58	M + H	Hypotaurine	C2H7NO2S	2.53	0.01
(9)	130.05	1.06	M + H	Pyroglutamic acid	C5H7NO3	2.30	0.05
(10)	526.29	10.27	M + Na	LysoPE(20:3(5Z,8Z,11Z)/0:0)	C25H46NO7P	2.24	0.02
(11)	450.36	10.44	M + Na	Stearoylcarnitine	C25H49NO4	1.56	0.03
(12)	482.33	10.11	M + H	LysoPC(15:0)	C23H48NO7P	1.38	0.04
(13)	127.04	4.14	M + Na	3-Hydroxybutyric acid	C4H8O3	1.36	0.02
(14)	217.10	4.70	M + Na	4-Aminohippuric acid	C9H10N2O3	1.26	0.03
(15)	570.36	10.89	M + Na	LysoPC(20:2(11Z,14Z))	C28H54NO7P	1.20	0.03
(16)	190.08	1.06	M + H	Kynurenic acid	C10H7NO3	1.01	0.00
(17)	540.33	10.57	M + FA − H	LysoPC(16:0)	C24H50NO7P	8.32	0.01
(18)	331.18	7.56	M + FA − H	Androstenedione	C19H26O2	4.11	0.02
(19)	507.21	6.53	M + FA − H	6-Dehydrotestosterone glucuronide	C25H34O8	3.36	0.03
(20)	283.12	6.58	M − H	p-Cresol glucuronide	C13H16O7	2.12	0.03
(21)	524.28	10.27	M + FA – H	LysoPE(18:1(9Z)/0:0)	C23H46NO7P	1.69	0.04
(22)	586.31	9.79	M + FA − H	LysoPC(20:5(5Z,8Z,11Z,14Z,17Z))	C28H48NO7P	1.61	0.02
